# Perioperative Antibiotic Choice Does Not Affect Wound Complications in the Operative Treatment of Ankle Fractures

**DOI:** 10.7759/cureus.73625

**Published:** 2024-11-13

**Authors:** John J Lee, Tinisha R Ricks, Sunakshi Garg, Jennifer O Alegun, Bohan Li, Lauren R Klein, Catherine G Caronia

**Affiliations:** 1 Orthopedic Surgery, Good Samaritan University Hospital, West Islip, USA; 2 Podiatry, Good Samaritan University Hospital, West Islip, USA; 3 Emergency Medicine, Good Samaritan University Hospital, West Islip, USA; 4 Pediatrics, Good Samaritan University Hospital, West Islip, USA

**Keywords:** ankle fracture, antibiotic prophylaxis, cefazolin, orif, perioperative antibiotics, surgical site infection

## Abstract

Background and aims

Ankle injuries are one of the most common lower extremity traumas reported, with nearly half needing surgical intervention. For those who undergo surgical treatment, surgical site infection (SSI) is not a rare complication. Cefazolin is the recommended preoperative surgical chemoprophylaxis for surgical site infection. This exploratory study sought to evaluate the efficacy of cefazolin prophylaxis at the time of primary open reduction and internal fixation (ORIF) for ankle fractures compared to the efficacy of non-cefazolin chemoprophylaxis with respect to wound complications.

Methods

A single-center retrospective study of adult patients who underwent open reduction and internal fixation (ORIF) of a closed ankle fracture between January 1, 2012, and April 11, 2023, was conducted at Good Samaritan University Hospital. Patients were compared based on the perioperative antibiotic received per our hospital’s guidelines: cefazolin (given by weight: 2 g < 120 kg and 3 g ≥ 120 kg) or a non-cefazolin alternative for a reported cefazolin or penicillin allergy (clindamycin 900 mg and/or vancomycin 1 g < 80 kg or 1.5 g ≥ 80 kg). Group 1 consisted of 132 patients who underwent ORIF of the ankle after having received cefazolin. Group 2 consisted of 19 patients who underwent ORIF of the ankle after having received a non-cefazolin antibiotic. The outcomes measured were postoperative infection, infection requiring surgery, and dehiscence. Associations between the American Society of Anesthesiologists (ASA) physical classification score, body mass index (BMI), and ankle fracture classification and our primary outcomes were also reviewed.

Results

Of the 151 patients, 22 patients were reported to have complications. Complications were defined as postoperative infection, infection requiring surgery, and dehiscence. There was no statistically significant difference in these complication rates (infection, p = 0.9; infection requiring surgery, p = 0.6; and dehiscence, p = 0.5) between the cefazolin and non-cefazolin cohorts. The average follow-up time after surgery in both groups was eight months.

Conclusions

There were no significant differences in complication rates between the cefazolin and non-cefazolin cohorts. In turn, prophylactic antibiotic type, among those reported in this study, does not appear to be a prominent influence on the rate of wound complications in ORIF of ankle fractures. The sample size of this study, however, is a major limitation. These results can help guide a larger study of the efficacy of antibiotic chemoprophylaxis choice in ankle ORIF surgeries.

## Introduction

Ankle fractures account for approximately 19.2% of all lower extremity fractures that present in emergency rooms in the United States every year and account for 7.6% of all fractures in adults [1,2]. The severity, mechanism of injury, fracture pattern, and patient factors typically dictate operative versus nonoperative treatment with nearly 50% of ankle fractures requiring open reduction and internal fixation (ORIF) [2]. For patients who undergo operative treatment, surgical site infection (SSI) is one of the most common and costly postoperative complications [3-5].

The Centers for Disease Control and Prevention (CDC) describes a surgical site infection as an infection of the operative area within 90 days after fracture surgery [6]. The reported risk factors for SSI after the operative treatment of ankle fractures include higher body mass index (BMI), American Society of Anesthesiologists (ASA) score greater than or equal to 3, diabetes, alcohol abuse, open fracture, joint subluxation/dislocation, the mechanism and grade of injury, and chronic heart disease [2,5]. Surgical site infections in the lower extremity can lead to an increased number of surgical procedures, an increased rate of return to the operating room, worse patient outcomes, and, in the case of severe infection, lower extremity amputation and permanent disability [2]. SSIs can also increase the cost of treatment by $4,500 to $15,000 or more [2].

Numerous approaches have been taken to minimize the rate of SSIs, such as preoperative skin preparation, prophylactic antibiotic therapy, and postoperative protocols. Previous studies have sought to define the optimal time frame in which to provide antibiotic therapy as SSI prophylaxis, with the consensus being 15 minutes to one hour prior to incision [7]. Patients who do not receive appropriate antibiotic prophylaxis are 2.32 times more likely to have an SSI [3]. Some of the most common organisms isolated from confirmed SSIs and deep surgical site infections following foot and ankle surgeries are methicillin-resistant and methicillin-susceptible *Staphylococcus aureus* and *Pseudomonas aeruginosa* [3,8].

According to the American Academy of Orthopaedic Surgeons (AAOS), intravenous (IV) cefazolin remains the choice of perioperative antibiotics for foot and ankle surgery. The AAOS’s evidence-based clinical practice guidelines recommend systemic antibiotic prophylaxis of 1-3 g of cefazolin administered within 60 minutes of surgical incision in patients undergoing surgery due to the trauma of a major extremity. While cefazolin may be recommended, in the case of patients with severe self-reported penicillin allergies (i.e., anaphylaxis) and those with a history of methicillin-resistant *Staphylococcus aureus* (MRSA), the use of cefazolin may be contraindicated or not ideal due to its inefficacy. For these patients, alternative antibiotic therapy with clindamycin and/or vancomycin is recommended, respectively [9-11].

The primary objective of the study was to determine the rates of surgical site infection and wound complications in those treated with cefazolin versus a non-cefazolin alternative. A secondary objective was to explore factors associated with an increased rate of SSI/wound complication.

## Materials and methods

Study overview

A single-center retrospective cohort pilot study of patients who underwent ORIF of the ankle between January 1, 2012, and April 11, 2023, was conducted.

Ethical considerations

The study protocol was reviewed by the Good Samaritan University Hospital Institutional Review Board (IRB) and determined to be an exempt human subjects’ research study. This research study complies with all regulations, and informed consent was obtained as required by 45 CFR 46.104.

Study procedure

Data were extracted from the electronic health record system. Medical records of patients who had undergone an open reduction and internal fixation (ORIF) for an ankle fracture were extracted utilizing the Epic SlicerDicer program (Madison, WI).

Study population and criteria

Patients 18 years and older who underwent operative treatment for a closed ankle fracture and were given perioperative antibiotics and who had a minimum follow-up of three months were included. Pediatric patients, patients not given perioperative antibiotics, patients who presented with open ankle fracture and tibial pilon fracture, polytrauma patients, those with less than three months of documented follow-up, and those with a history of previous lower extremity wounds or surgical site infection were excluded. Pediatric patients were excluded as the focus of our study was on ankle fractures with closed physes. Open fractures and those with a history of lower extremity wounds or surgical site infection were excluded as their surgical site infection rates are substantially higher than those of closed fractures and those without a history of wounds and infections.

Study assessments

The primary outcomes measured were wound complication rates for patients treated with cefazolin versus non-cefazolin alternative antibiotics. Wound complications were defined as infection following open reduction and internal fixation (ORIF), infection requiring a return to the operating room, and wound dehiscence (the separation of surgically approximated wound edges) within 90 days of open reduction and internal fixation. The association between the American Society of Anesthesiologists physical classification score (ASA score), body mass index (BMI), and ankle fracture classification and wound complications was also reviewed.

Statistical analysis

Baseline demographics were analyzed descriptively, including means with standard deviations (SD) and medians with interquartile ranges (IQRs), as appropriate. Variables are presented for the entire cohort and then stratified by antibiotic choice (cefazolin versus non-cefazolin cohorts). For each group, demographic variables were compared utilizing a chi-squared test for proportions for count data and a Mann-Whitey U test for nonparametric continuous data with an alpha of 0.05 considered significant.

Outcome data (infection not requiring surgery, infection requiring surgery, and wound dehiscence) are presented as point estimates with associated 95% confidence intervals (CI) to demonstrate effect size. We also report a composite outcome for infection with or without surgery, also with a 95% confidence interval.

To compare outcomes for each group, we report a difference in proportions with associated 95% confidence intervals. We also described outcome differences between groups as an unadjusted odds ratio (uOR), depicting the odds for each outcome based on antibiotic choice.

A multivariable logistic regression exploring variables associated with increased odds of infection with antibiotic choice as the primary independent variable of interest was planned; however, due to the small sample size and an outcome count of <10, this was not pursued, as such a multivariable model would not be valid given these constraints based on established model assumptions.

## Results

**Figure 1:**
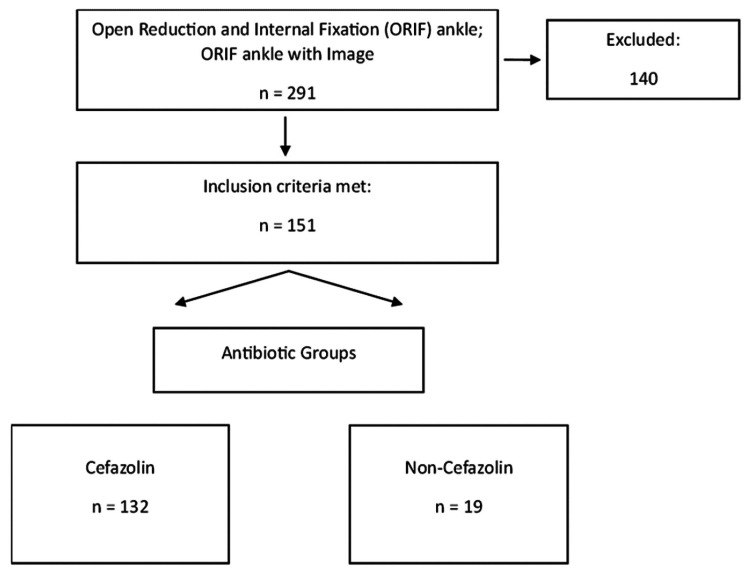
Flow diagram of patient selection

A review of the medical records of patients who had undergone an open reduction and internal fixation (ORIF) yielded a total of 291 patients (Figure 1).

One hundred forty patients were excluded from the study as they did not meet the inclusion criteria (10 were pediatric patients, three had no preoperative antibiotics given, 15 were open ankle fractures, five were tibial pilon fracture, 11 were polytrauma patients, six had previous wound complications, and 90 had less than three months of follow-up). The remaining 151 patients represented the two groups of interest. Group 1 consisted of one hundred thirty-two patients who underwent ORIF of the ankle after having received cefazolin. Group 2 consisted of 19 patients who underwent ORIF of the ankle after having received a non-cefazolin antibiotic alternative. The most common antibiotic alternative used was vancomycin (95%). At our institution, vancomycin is used as the first alternative to cefazolin.

Group 1 had 55 men and 77 women, and group 2 had three men and 16 women. The mean age (SD) at the time of the procedure in group 1 was 53 (15) years (range: 25-90) and 61 (17) (range: 22-84) in group 2 (p = 0.6). Between both groups, the median ASA class at the time of the procedure was class 2, mild systemic disease (range: 2-3). BMI was 30 (six) (range: 17-60.19) in group 1, compared to 29.1 (nine) (range: 22-45) in group 2 (p = 0.3) (Table 1).

**Table 1:** Patient characteristics seen in ORIF of ankle fractures receiving cefazolin versus those receiving non-cefazolin antibiotics *American Society of Anesthesiologists (ASA) physical classification score IQR, interquartile range; BMI, body mass index; ORIF, open reduction and internal fixation

	Group 1: cefazolin (n = 132)	Group 2: non-cefazolin (n = 19)	P-value
Age	53 ± 15	61 ± 17	0.6
Male (%)	55 (42)	3 (16)	0.03
ASA (IQR)*	2 (2-3)	2 (2-3)	0.4
BMI	30 ± 6	29.1 ± 9	0.3
Fracture type (%)			0.2
Bimalleolar	50 (38)	5 (26)	
Trimalleolar	48 (36)	12 (63)	
Distal fibula	22 (17)	2 (10)	
Distal tibia	5 (4)		
Syndesmosis	7 (5)		

The preoperative description of the ankle fracture mechanism included bimalleolar, trimalleolar, isolated fibular, isolated distal tibia, and isolated syndesmotic injuries. Bimalleolar ankle fractures were present in 50 (38%) patients in group 1 compared to five (26%) patients in group 2. Trimalleolar ankle fractures were present in 48 patients (36%) in group 1 compared to 12 patients (63%) in group 2. Isolated distal fibula fracture was present in 22 (17%) patients in group 1 compared to two (10%) patients in group 2. Isolated distal tibia and isolated syndesmotic injury were only described in group 1 in five patients (4%) and seven patients (5%), respectively.

**Figure 2:**
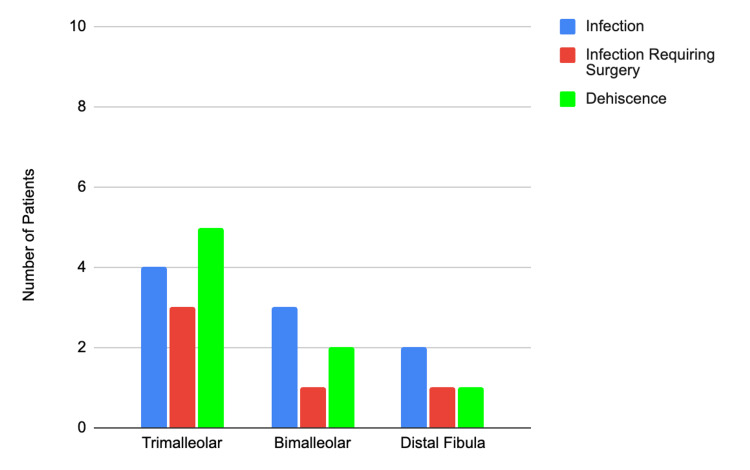
Summary of patient complications based on fracture pattern Total number of patients grouped by fracture pattern

Twenty-two patients (15%) went on to have complications. Complications were defined as infection, infection requiring surgery, and wound dehiscence (Table 2). Twelve patients (9%, 95% CI: 5-15) in group 1 and two patients (11%, 95% CI: 1-33) in group 2 (p = 0.9) had a postoperative infection. Four patients (3%, 95% CI: 1-8) in group 1 and one patient (5%, 95% CI: 1-26) in group 2 had postoperative infection requiring surgery. No difference was found between the groups with respect to wound dehiscence, with seven patients (5%, 95% CI: 2-11) in group 1 and one patient (5%, 95% CI: 1-26) in group 2 (p = 0.6). Complications were also summarized based on the ankle fracture mechanism for those fractures involving the fibula (Figure 2). Those with infections not requiring surgery (n = 9) necessitated either oral (5/9) or intravenous (4/9) antibiotics as treatment. Among those infections requiring surgery (n = 5), four had IV antibiotics, and one had oral antibiotics following their surgery for infection treatment.

**Table 2:** Analysis of surgical complications All data presented as N with % and a 95% confidence interval (CI) of the proportion *Cefazolin is the index group for the unadjusted OR (uOR) OR: odds ratio

	Cefazolin (N = 132)	Non-cefazolin (N = 19)	Difference in proportion (95% CI)	Unadjusted OR* (95% CI)
Infection	12 (9%, 5-15)	2 (11%, 1-33)	2% (-8-23)	0.9 (0.2-4.1)
Infection requiring surgery	4 (3%, 1-8)	1 (5%, 1-26)	2% (-4-21)	0.6 (0.1-5.3)
Infection not requiring surgery	8 (6%, 3-12)	1 (5%, 1-26)	-1% (-7-18)	1.2 (0.1-9.8)
Wound dehiscence	7 (5%, 2-11)	1 (5%, 1-26)	0% (-7-19)	1.0 (0.1-8.6)

The total number of patients grouped by fracture pattern is as follows: trimalleolar, 60; bimalleolar, 55; and distal fibula, 24.

There was no difference in total follow-up time between those receiving cefazolin and those receiving non-cefazolin antibiotics (p = 0.5). Between groups, the average follow-up time after surgery was eight months with a range of 3-36 months in group 1 and 3-27 months in group 2.

## Discussion

Surgical site infection (SSI) is one of the most common complications following the operative treatment of ankle fractures. In hospitalized patients, SSIs account for nearly half (40%) of all healthcare-associated infections [12]. Current evidence-based guidelines recommend cefazolin as the perioperative gold standard to reduce the risk of SSI. The recommendation is due to the magnitude of its coverage (common skin flora, methicillin-susceptible *Staphylococcus aureus* {MSSA}, and *Streptococcus* species), its bactericidal activity, and its ability to reach optimal antibacterial concentration in tissues rapidly and penetrate the bone [12]. However, patients with documented or self-reported penicillin allergies often receive non-cefazolin antibiotics, which have perceived comparable efficacy. In our study, the most used non-cefazolin antibiotic was vancomycin. A recent study comparing the SSI rate in lower extremity joint replacements in patients who had cefazolin or a non-cefazolin alternative demonstrated a 50% higher rate of SSI in the non-cefazolin alternative group [9]. This draws concerns about the actual efficacy of the non-cefazolin antibiotics. Another drawback associated with using alternative antibiotics is the various potential adverse effects noted, including ototoxicity and nephrotoxicity from vancomycin [12].

The primary focus of this retrospective cohort study was to investigate if non-cefazolin antibiotic regimens given perioperatively were associated with an increased rate of SSI or wound complications following ORIF of ankle fractures. Our results suggest that the choice of antibiotic is not a statistically significant contributor to the complication rate of ORIF of ankle fractures. Wyles et al. demonstrated a decreased risk of prosthetic joint infection with the use of cefazolin when compared to antibiotics such as vancomycin and clindamycin [9]. A major limitation of our study is our sample size, which may lead to a type 2 error. In Wyles et al.’s study, 22,705 patients were included over a more than 13-year period [9].

We also examined previously identified risk factors [2,3,8,11,13]. We were unable to identify a statistically significant association between ASA, BMI, or ankle fracture pattern and the development of a wound complication. This is likely because the risk of developing an SSI is multifactorial within the patient population, and our small sample size is better suited for inferential comparisons. Hansen et al. investigated the risk factors contributing to complications after foot and ankle surgery. They demonstrated patients with an ASA of 2 or higher were at an increased risk of complications [1]. Our cohorts had an average ASA score of 2, with a range of 2-3, in line with previously reported studies. Tantigate et al. demonstrated that when comparing groups with SSI and non-SSI, the only significant independent predictors were prolonged surgery and nonambulatory surgery and that 91.8% of the risk of an SSI can be predicted by ASA score and the duration of surgery [7]. Although not statistically significant, there was a trend noted in our results between fracture pattern and SSI. Trimalleolar injuries tend to be more severe than bimalleolar and single malleolar injuries in terms of tissue damage. In this study, SSI was seen most frequently in patients with a trimalleolar fracture pattern. In addition, Ovaska et al. determined that fracture patterns can determine the risk of infection requiring surgery. They proposed that Danis-Weber type C fractures are at higher risk for infection requiring surgical debridement [14]. This is supported by our results, which indicated that the highest number of infections necessitating a return to the operative room was in trimalleolar fractures.

As stated prior, a major limitation of this study is the sample size. All consecutive patients were included, yet the study was inherently underpowered due to available cases. There are no prior ankle fracture studies that we are aware of that have looked into our hypothesis. Multiple surgeons performed different procedures with different operative techniques and fixation methods over the study period. Smith and Hing determined that ankle surgeries necessitating the use of a tourniquet had higher rates of wound complications and deep vein thrombosis incidence [15]. Butterworth et al. determined that SSI in foot and ankle surgery was linked to surgeon experience, with the more senior surgeons having lower complication rates [13].

To our knowledge, this is the first study of its kind to examine perioperative antibiotic choices in patients undergoing ORIF of ankle fractures. Previous studies on antibiotic choice and infections have focused on hip and knee joint replacement surgeries where it seems to have an effect. We believe that this adds to the body of knowledge of antibiotic influence on surgical site infections in ankle fractures and may guide surgeons in clinical decision-making when a cefazolin antibiotic is contraindicated. Further studies could benefit from a prospective viewpoint and larger sample sizes to make more complete generalizations.

## Conclusions

Our study suggests that perioperative antibiotic choice between cefazolin and a non-cefazolin alternative may not affect SSI or wound complication rates in ankle fracture patients treated operatively. Our findings are limited by its small sample size. A larger study appropriately powered with parameters used from this study is needed.
